# Bochdalek hernia in an adult: two case reports and a review of perioperative cardiopulmonary complications

**DOI:** 10.1186/s40792-020-00833-w

**Published:** 2020-04-17

**Authors:** Masayuki Akita, Nobuaki Yamasaki, Taiichiro Miyake, Kazuya Mimura, Eri Maeda, Tohru Nishimura, Koichiro Abe, Akihito Kozuki, Kunio Yokoyama, Hiroaki Kominami, Tomohiro Tanaka, Manabu Takamatsu, Kunihiko Kaneda

**Affiliations:** Department of Surgery, Kakogawa Central City Hospital, Kakogawa, 675-8611 Japan

**Keywords:** Bochdalek hernia, Adult, Pulmonary complications

## Abstract

**Background:**

Bochdalek hernia in an adult is very rare and often needs an immediate surgical repair for the herniation. Although its etiology and surgical techniques have frequently been reported, perioperative complications, especially cardiopulmonary problems, remain unknown. We reported two adults with Bochdalek hernia and reviewed the published literatures with a focus on these issues.

**Case presentation:**

We experienced two adult cases of Bochdalek hernia with gastrointestinal strangulation. One case had massive herniation of the stomach, colon, spleen, and pancreas in the left chest, causing repeated vomiting. The other had a right-side hernia with strangulation of the colon. We successfully performed emergency repairs of these diaphragmatic hernias without any postoperative complications.

**Conclusions:**

Our literature review revealed that life-threatening cardiopulmonary complications, such as empyema or cardiac arrest caused by the tamponade effect of the herniated viscera, sometimes occurred in patients with Bochdalek hernia. These complications were found in Bochdalek hernia with gastrointestinal strangulation.

## Background

Bochdalek hernia is a congenital diaphragmatic defect caused by a failure in posterolateral diaphragmatic formation and is associated with severe pulmonary complications during perinatal life. However, some patients with this hernia are asymptomatic and exhibit respiratory and/or abdominal symptoms caused by the herniation of abdominal viscera for the first time in adulthood [1–3].

In the last decade, there have been more than 50 cases of Bochdalek hernia in adults [4–57]. Surgical approaches have varied between institutions: laparoscopy or thoracoscopy as well as primary closure or mesh repair. Although most cases had a favorable postoperative course, some developed severe cardiopulmonary complications and these risk factors remain unclear.

We encountered two adult cases of Bochdalek hernia. One case had massive herniation of the stomach, colon, spleen, and pancreas in the left chest with gastrointestinal obstructive symptoms, and the other had a right-side hernia with strangulation of the colon. We herein described these cases and reviewed previous case reports, particularly those with a focus on respiratory complications.

## Case presentation

### Case 1

**Fig. 1:**
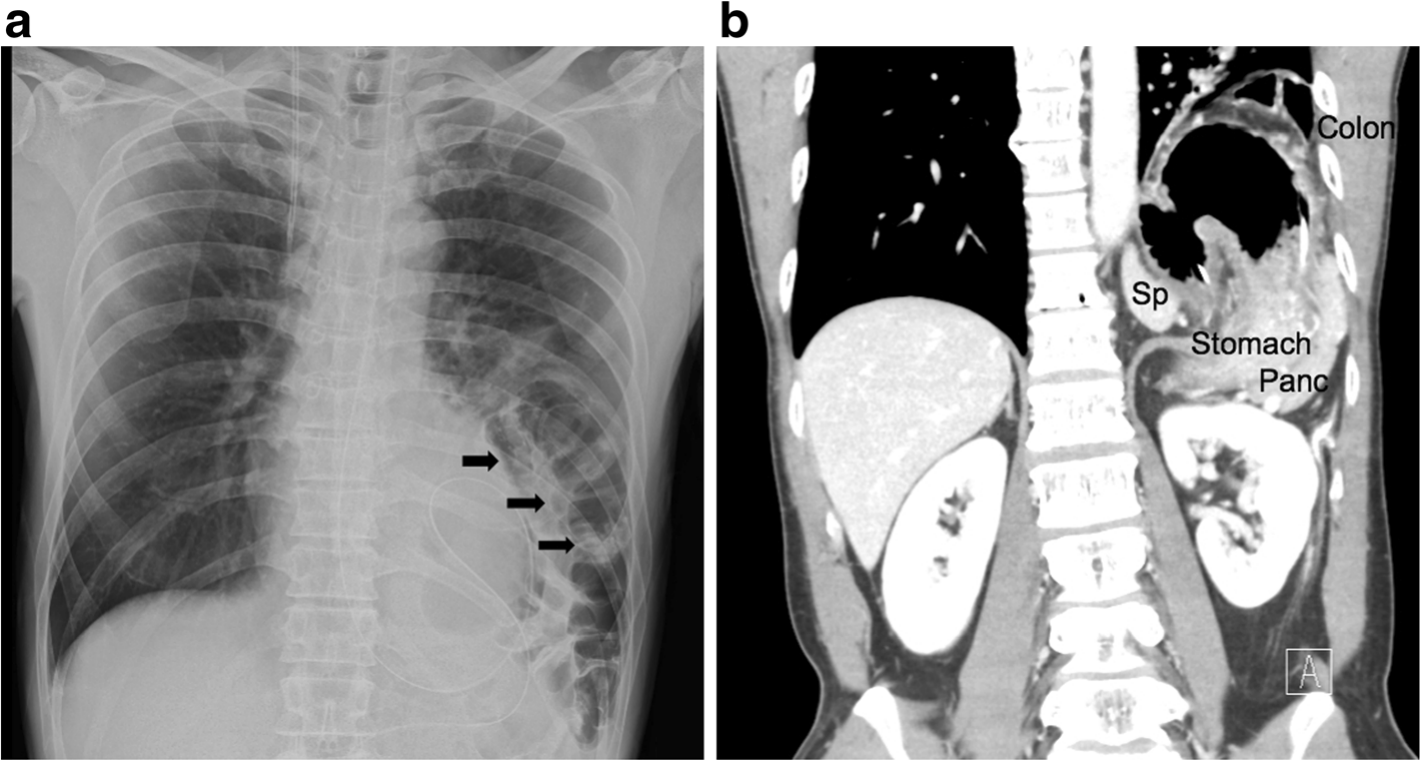
Chest X-ray showing colon gas and a nasogastric tube above the left diaphragm. Arrows showed the herniated colon (**a**). Coronal CT imaging revealed herniation of the stomach, pancreas, spleen, and colon (**b**)

**Fig. 2:**
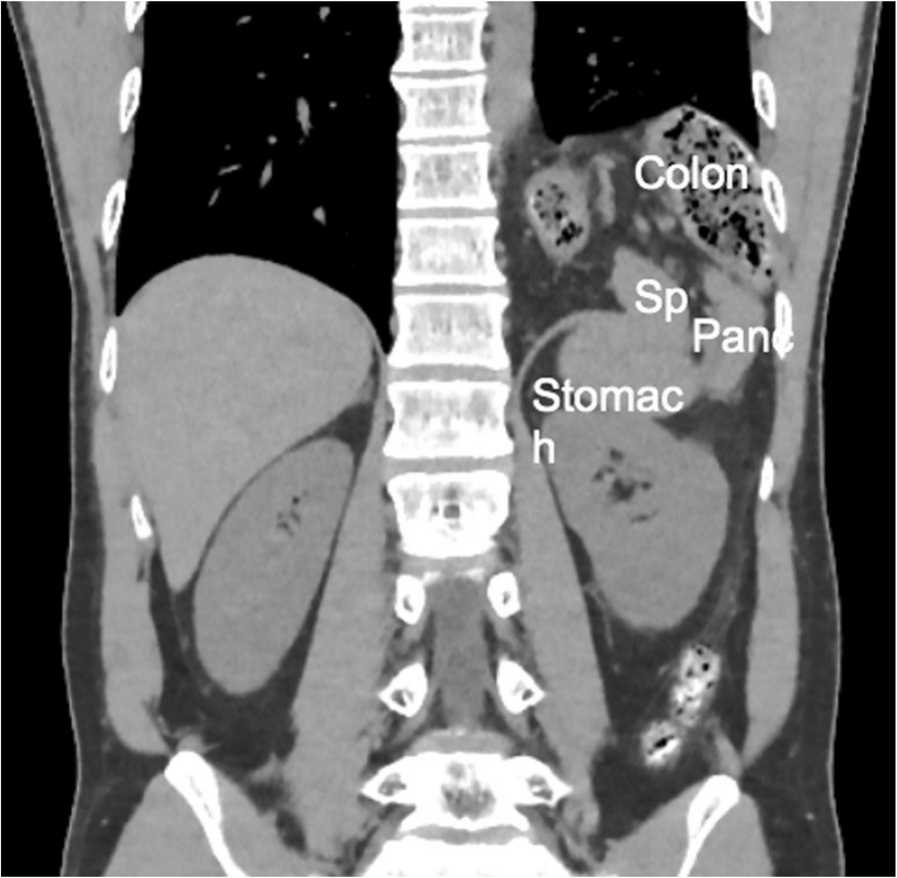
CT imaging 10 years before surgery showed modestly prolapsed viscera (colon, spleen, and pancreas)

A 43-year-old healthy male was admitted with abdominal pain and vomiting for 3 days. He had a previous history of a hemorrhagic gastric ulcer 10 years before and no trauma. X-ray and computed tomography (CT) revealed a left diaphragmatic hernia (Fig. 1a, b). CT showed that the entire stomach, spleen, pancreatic tail, and splenic flexure occupied two thirds of the left thoracic cavity. Upside-down stomach and gastric volvulus causing vomiting were detected. The patient was diagnosed with Bochdalek hernia based on the presence of a hernia orifice in the posterolateral aspect of the left diaphragm. When CT imaging performed 10 years before was retrospectively reviewed, a more modest degree of Bochdalek hernia was detected, which may have caused the gastric ulcer at that time (Fig. 2).

A nasogastric tube was inserted to decompress the stomach; however, the patient had persistent abdominal pain and incarcerated obstruction. He was referred to our hospital for a surgical intervention. The results of laboratory examinations were within normal ranges, and the respiratory function test showed a percentage of vital capacity of 93.9% and forced expiratory volume in 1 s of 106.2%. We preoperatively discussed with an anesthesiologist about perioperative administration of neutrophil elastase inhibitor or steroids for re-expansion pulmonary edema which we have expected to occur after restoring herniated viscera.

**Fig. 3:**
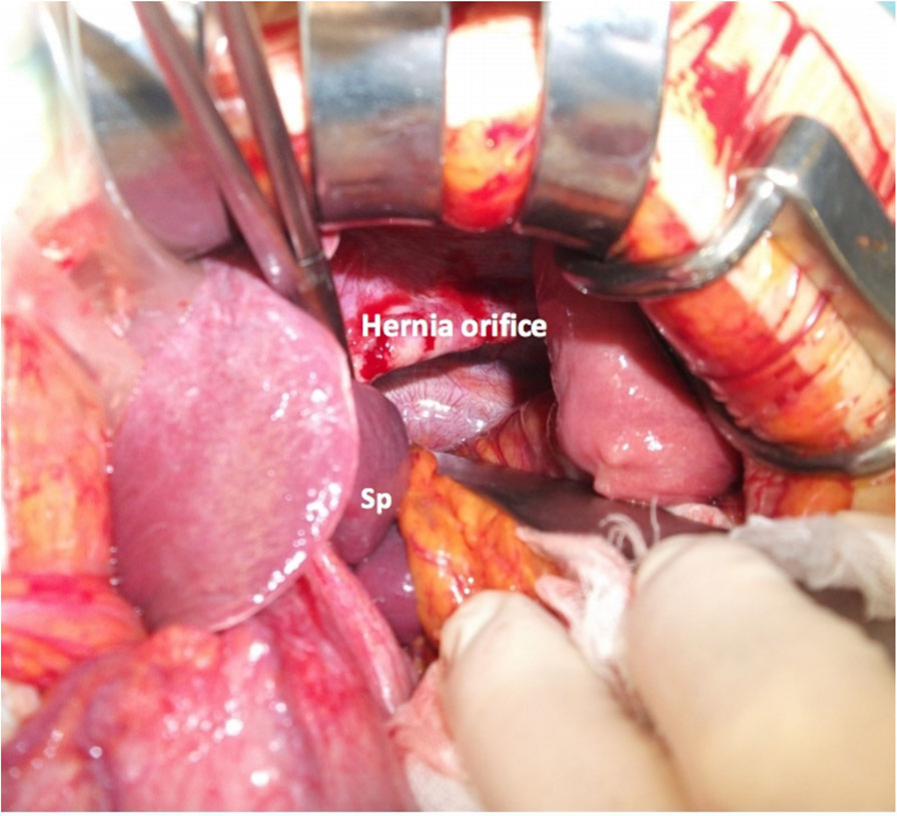
After reducing the herniation. The hernia orifice was 7 cm, and there was no hernia sac

**Fig. 4:**
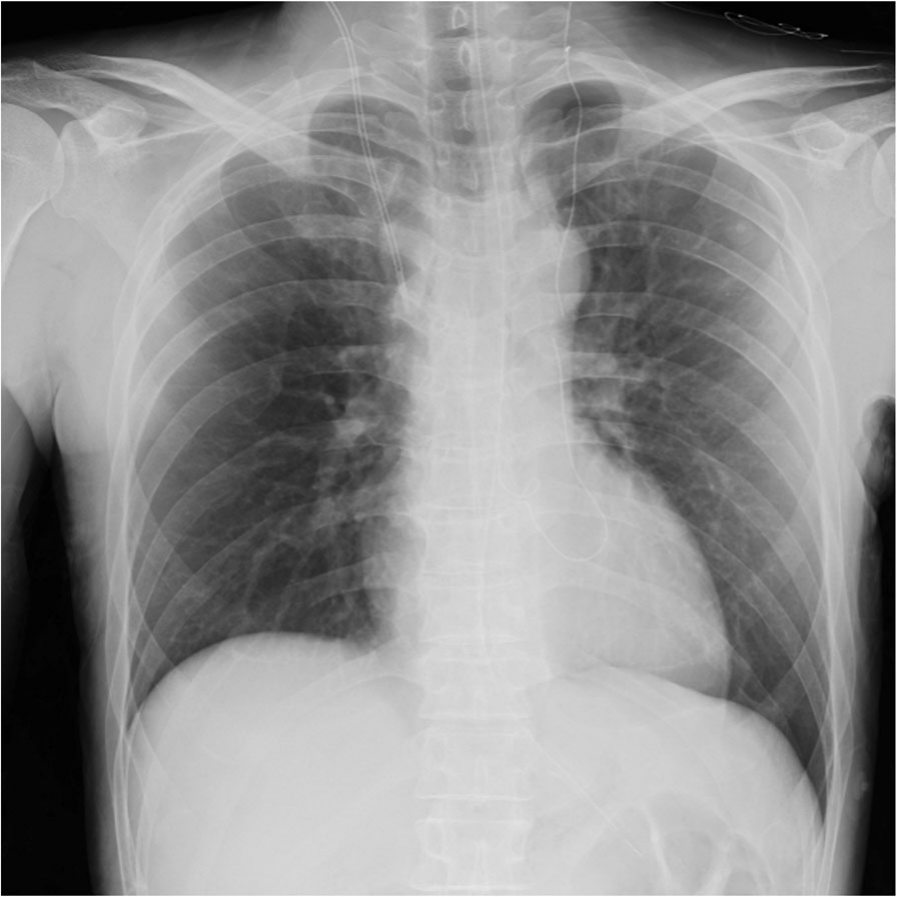
Chest X-ray 5 h after surgery showing a fully expanded left lung without re-expansion pulmonary edema

The patient underwent L-shaped laparotomy to surgically repair Bochdalek hernia. A 7-cm hernia orifice was observed in the left posterior diaphragm (Fig. 3). Herniated viscera were carefully returned back into the abdominal cavity, and a hernia sac was not detected. There were no adhesions between abdominal viscera and the thoracic cavity or left lung. The left diaphragmatic defect was repaired by primary closure with 0 absorbable thread without diaphragmatic tension. Chest X-ray immediately after surgery showed a fully expanded left lung and that 5 h after surgery showed no pleural effusion or re-expansion pulmonary edema (Fig. 4).

The postoperative course was uneventful, and diaphragmatic hernia has not recurred for 3 months.

### Case 2

**Fig. 5:**
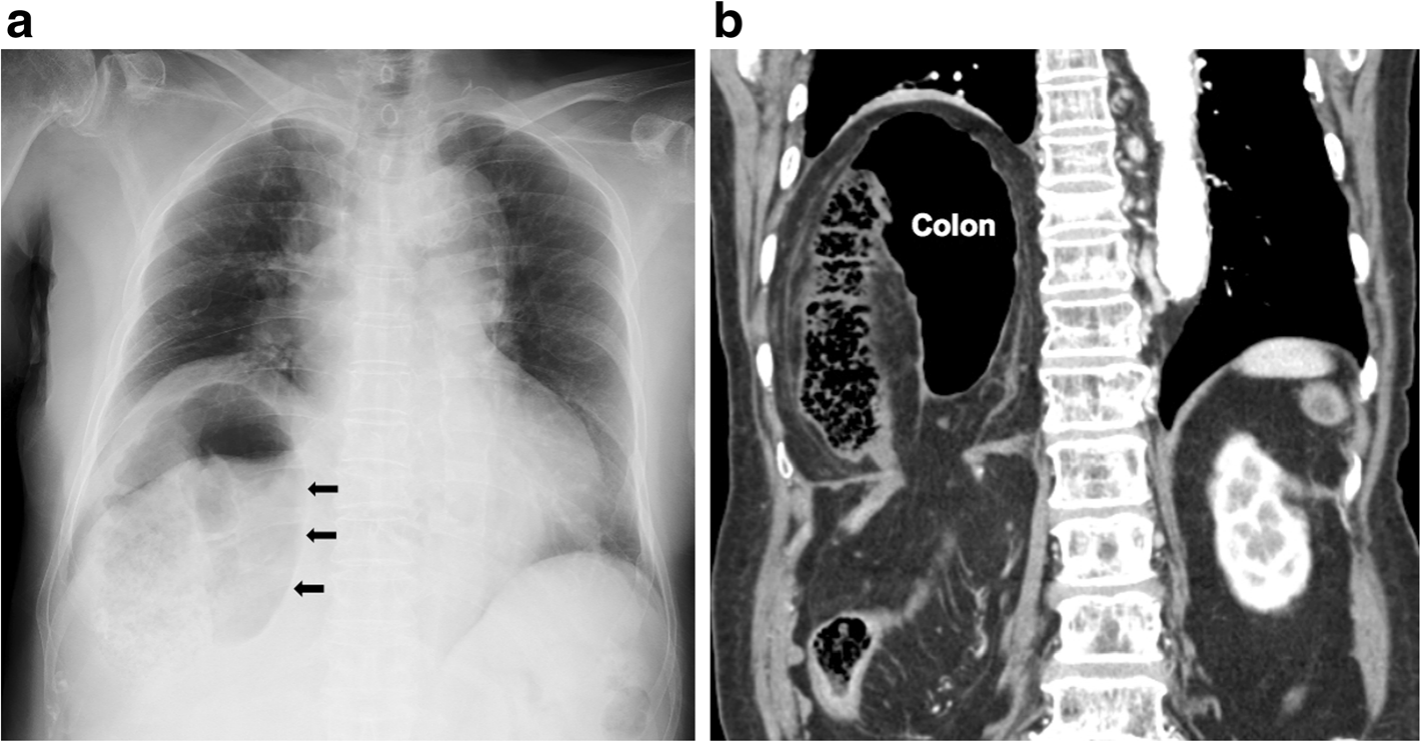
Chest X-ray showing the dilated colon above the right diaphragm (**a**). CT scan showing strangulation of the colon with some ascites fluid in a sac (**b**)

The patient was an 87-year-old female with a previous history of an asymptomatic diaphragmatic hernia. She was transferred to our hospital with sudden abdominal pain and vomiting. X-ray and CT imaging showed a right diaphragmatic hernia with a dilated transverse colon and modest ascites (Fig. 5a, b). Since the hernia orifice was located in the posterolateral aspect of the right diaphragm, she was diagnosed with Bochdalek hernia with incarceration of the transverse colon. The results of laboratory examinations and arterial blood gas measurements were within normal ranges, with the exception of a modest oxygen level (50.6 mmHg).

She underwent emergency reverse L-shaped laparotomy for strangulated Bochdalek hernia. A 4-cm hernia orifice was observed in the right posterior diaphragm. The right transverse colon was incarcerated into the right chest cavity, and the prolapsed colon was returned to the abdominal cavity. A hernia sac was detected as well as adhesions between the prolapsed colon and hernia sac. The hernia orifice was repaired by a primary suture with 0 absorbable thread. The necrotic colon was excised by right hemicolectomy, and a thoracostomy tube was inserted into the right chest. She recovered without any complications, and there was no recurrence in the month following surgery.

## Discussion

Bochdalek hernia is a well-known congenital diaphragmatic disease due to life-threatening lung hypoplasia and pulmonary hypertension during perinatal life. It is caused by a diaphragmatic malformation in the posterolateral side. However, some cases of Bochdalek hernia remain asymptomatic until adulthood [1–3]. Previous reviews described Bochdalek hernia in adults, with the most common symptoms at presentation being chest and/or abdominal pain (66%) and symptoms of ileus (38%) [3]. Surgical repair for herniation by transthoracic or transabdominal approaches is recommended for symptomatic patients with prolapsed viscera. Although the etiology of Bochdalek hernia and surgical techniques have frequently been reported, perioperative cardiopulmonary pitfalls remain unknown.

Between 2010 and 2018, 55 operated case reports were obtained in PubMed/Medline with the keywords of “Bochdalek hernia” and “adult” [4–57]. Table 1 summarizes the characteristics of these reports and the present cases. The mean age of patients was 41 years, and left-side Bochdalek hernia was more common (*n* = 36, 65%). The majority of patients presented with chest and/or abdominal symptoms (*n* = 53, 96%), such as pain and dyspnea. A hernia sac was absent in 62% (present in 13 patients). Surgical approaches were as follows: thoracoscopy (*n* = 2), thoracostomy (*n* = 8), laparoscopy (*n* = 19), and laparotomy (*n* = 26).

**Table 1:** Clinical features of 55 case reports and our cases

	Age	Sex	Chief complaint	Hernia orifice (cm)	Hernia sac +/−	Surgical procedure	Postoperative complications, ≥ C-D IIIa
Previous all cases, *n* = 55	41 (26–63)	Male, *n* = 22 Female *n* = 35	Abdominal pain, *n* = 26 Chest pain, *n* = 7 Dyspnea, *n* = 13 Vomiting, *n* = 2	7 (5–10)	13/21	Thoracoscopy, *n* = 2 Thoracotomy, *n* = 8 Laparoscopy, *n* = 19 Laparotomy, *n* = 26	Pleural effusion, *n* = 1 Pneumonia, *n* = 2 Empyema, *n* = 4
Cases with gastrointestinal strangulation, *n* = 14^*^	41 (30–65)	Male, *n* = 5 Female, *n* = 9	Abdominal pain, *n* = 12 Chest pain, *n* = 0 Dyspnea, *n* = 1 Vomiting, *n* = 1	6 (4.5–8)	7/7	Laparoscopy, *n* = 2 Laparotomy, *n* = 12	Pneumonia, *n* = 1 Empyema, *n* = 4
Case 1	50	M	Abdominal pain Vomiting	7	Absent	Laparotomy	None
Case 2	87	F	Abdominal pain	4	Present	Laparotomy	None

Continuous variables are shown as a median (quartile)

^*^Stomach: *n* = 7; small intestine: *n* = 6; colon: *n* = 2

In terms of preoperative cardiopulmonary problems, 3 patients were initially misdiagnosed with pneumothorax with air in the chest cavity on imaging tests [4, 32, 40]. A severely dilated stomach or colon in the chest mimicked a pneumothorax on chest X-rays, and 2 patients underwent thoracic drainage. Seventeen cases (31%) showed a mediastinal shift toward the opposite side on X-rays, and cardiac arrest occurred in one case, which may have been caused by the tamponade effect of the hugely dilated intrathoracic stomach [24]. Another patient with preoperative cardiac arrest caused by the hemorrhagic shock had herniation of the stomach and spleen [14]. The entire stomach was necrotic, and there was a large amount of bleeding from the spleen.

Intraoperatively, adhesions between the herniated viscera and a sac or the lungs were often observed (*n* = 6, 11%), and adhesiolysis was safely performed by both abdominal and thoracic approaches [8, 20, 21, 34, 55, 57]. Gastrointestinal strangulation was detected (stomach: *n* = 7; small intestine: *n* = 6; colon: *n* = 2), and their perforation frequently occurred (*n* = 5).

Postoperatively, some patients needed respiratory support, including mechanical ventilation or pleural effusion puncture (*n* = 7) [10, 11, 32, 37, 43, 50, 53]. The most severe respiratory complication was empyema (*n* = 4), which led to the death of one patient [8, 39, 42, 54]. Although most patients with Bochdalek hernia had an uneventful postoperative course, empyema occurred in patients with ischemic or necrotic changes in the herniated abdominal viscera (Table 1). A thoracostomy tube may need to be intraoperatively inserted in patients with gastrointestinal necrosis for prevention of these respiratory complications.

We had concerns about intra- and postoperative re-expansion pulmonary edema after reducing the herniation, which is often observed after thoracic drainage for a pneumothorax. And we were perioperatively ready to administer neutrophil elastase inhibitor or steroids for re-expansion pulmonary edema. However, it did not occur in either patient during their postoperative courses. To the best of our knowledge, there has been no report on re-expansion pulmonary edema after surgery for Bochdalek hernia. One of the possible explanations for this is that the lungs of patients with Bochdalek hernia are often hypoplastic and postoperative overexpansion of the remnant lung may fill in the gaps; however, the lungs of patients with a pneumothorax may suddenly collapse and re-expand after drainage.

## Conclusions

Bochdalek hernia in adulthood is rare and has an acute course. Severe pulmonary complications are expected to occur especially in patients with gastrointestinal strangulation.

## Data Availability

Not applicable

## Abbreviation

CT: Computed tomography
